# Pertussis Infection and Bilateral Optic Neuropathy Leading to Blindness in a 68-Year-old Male Patient: Possible Link or a Coincidence?

**DOI:** 10.7759/cureus.101681

**Published:** 2026-01-16

**Authors:** Husam Jamil, Mahsa Momeni, Helen Devonport

**Affiliations:** 1 Acute Internal Medicine, Health Education Yorkshire and Humber, Wakefield, GBR; 2 Acute Medicine, Leeds Teaching Hospitals NHS Trust, Leeds, GBR; 3 Ophthalmology, Bradford Teaching Hospitals NHS Foundation Trust, Bradford, GBR

**Keywords:** bilateral optic neuropathy, blindness, bordetella pertussis, case report, pertussis vaccination

## Abstract

Whooping cough is a deadly infection that mainly targets infants, but its incidence is also on the rise in adults. Bilateral optic neuropathy is rare and can be due to genetic factors, infections or autoimmune disorders. We present a case of a 68-year-old man who presented to a UK hospital with worsening vision in both eyes for three days. His vital signs including blood pressure were within normal limits. He was reviewed by acute internal medicine, ophthalmology and neurology teams. His investigations were unremarkable except serological evidence of recent pertussis infection. Despite steroid therapy, his vision did not improve and he lost his vision. There is no case in the literature suggesting the link between pertussis and bilateral optic neuropathy in adults, making this case a possible first case report suggesting a link between pertussis infection and bilateral optic neuropathy in adults and hence warranting further studies.

## Introduction

Bordetella pertussis causes a respiratory infection known as 'whooping cough' and is a gram-negative coccobacillus [1]. Pertussis continues to circulate globally, with outbreaks occurring every 3 to 4 years, largely due to waning immunity. The reason for this waning immunity is that both natural infection and vaccination are ineffective in providing lifelong protection [1]. As a result, the disease burden has shifted from children to adults [2].

Bilateral optic neuropathy is rare [3] and has many established causes [4]. These include ischemia, inflammation, toxins, autoimmune and genetic factors. Symptoms can include sudden vision loss, painful movement of eyes and loss of colour perception. The link between bilateral optic neuropathy and pertussis infection remains unclear and is not documented in the literature. 

We present a case of bilateral optic neuropathy in a 68-year-old adult who had a recent pertussis infection.

## Case presentation

A 68-year-old gentleman presented to a UK hospital with a three-day history of blurred vision in both eyes following a referral by an optician, who had detected bilateral papilledema. The patient had a recent bout of ‘unusual cough’ which lasted a week, and he did not seek medical attention for it. His past medical history included hypertension, which was under good control, and for which he was on amlodipine, varicose vein surgery 12 years ago and a prolonged course of antibiotic treatment for a bilateral otitis externa infection three months prior to the presentation. He did not undergo any recent vaccination.

He was first seen in the same-day emergency care unit of the hospital from where an ophthalmology referral was made. His vital signs showed a blood pressure of 120/79, heart rate of 90 beats per minute, temperature of 36.5 degrees centigrade and oxygen saturation of 98% on room air. 

Upon ophthalmology review, visual acuity was 6/30 in the right eye and 6/36 in the left eye. Colour vision was tested with Ishihara plates, which was 1/13 in each eye; there was no RAPD (rapid afferent pupillary Defect), visual fields were full on confrontation, and extraocular movements were full range. On slit lamp examination, the anterior segment was unremarkable, and the posterior segment showed bilateral gross disc swelling with elevation of all disc margins as well as the cup, obscuration of major disc vessels, a circumferential halo and a splinter haemorrhage at the inferior disc margin. Optical coherence tomography (OCT) showed intraretinal fluid tracking down from the disc to the macula, worse in the left eye.

Over the course of the following six days, his visual acuity reduced down to light perception in each eye, and he developed a left RAPD. Slit lamp examination showed worsening disc swelling in both eyes. OCT showed intraretinal and subretinal fluid tracking down along the nasal disc margin as well as temporally up to the fovea.

**Figure 1:**
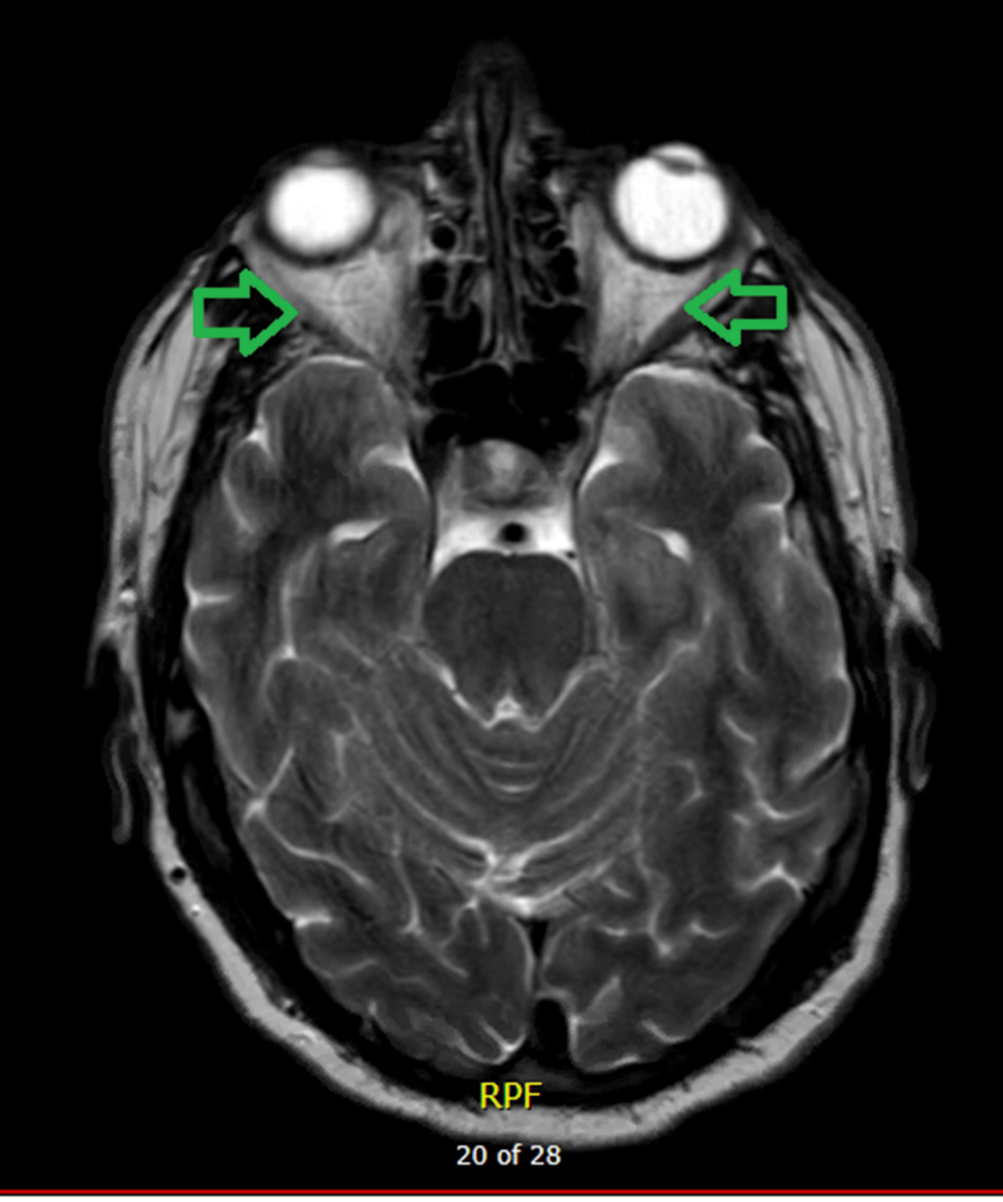
A normal MRI scan of the brain showing no abnormalities within the optic nerves bilaterally (green arrows).

The patient underwent a CT scan of the head, CT venogram, and CT scan of the thorax, chest and abdomen, all of which were normal. The MRI scan of the brain (Figure 1) did not show any abnormality in any of the intracerebral domains or optic nerves.

Blood and CSF investigations are summarised in Table 1. Blood tests, including Quantiferon TB test, liver function tests, viral PCR, thyroid function test, complement levels, anti MOG (myelin oligodendrocyte glycoprotein) and aquaporin 4 antibodies, were normal (the latter from CSF as serum). CSF was unremarkable. Interestingly, serum pertussis anti-PT IgG was markedly raised, signifying recent pertussis infection.

**Table 1:** Summary of key blood and cerebrospinal fluid investigations. IgG: Immunoglobulin G; PCR: Polymerase Chain Reaction; TB: Tuberculosis; TSH: Thyroid-Stimulating Hormone; n/a: Not Applicable; MOG: Myelin Oligodendrocyte Glycoprotein; IU/ml: International Units per Millilitre; mcg/L: Microgram per Litre; mU/L: Milliunits per Litre; mg/dL: Milligram per Decilitre; µL: Microlitre

Investigations	Results	Normal Range
Serum Pertussis anti-PT IgG	Greater than 300 IU/ml	0 - 70 IU/mL
Quantiferon TB test	5 mcg/L	1 - 10 mcg/L
COVID PCR	Negative	n/a
TSH	1.5 mU/L	0.4-40 mU/L
C3 levels	110 mg/dL	90-180 mg/dL
C4 levels	35 mg/dL	20-50 mg/dL
Anti MOG IgG	not detected	n/a
CSF aquaporin 4 antibodies	not detected	n/a
CSF protein	45 mg/dL	15 - 45 mg/dL
CSF glucose	50 mg/dL	45 - 80 mg/dL
CSF red cell count	3 per µL	upto 5 per µL
CSF white cell count	2 per µL	upto 5 per µL

He was treated jointly by the acute medicine, ophthalmology and the neurology teams as an inpatient. He received high-dose intravenous methyl prednisolone for three days, 1gm once daily and he was then switched to oral steroids. However, he did not make any significant recovery from his bilateral visual loss despite some improvement in disc swelling. 

He moved to a different region of the UK; hence, further auto-immunology tests were not done. His medical information was passed on to the local doctors of that region for follow-ups. He was counselled about probable post-infective optic neuropathy and registered as a blind person. 

## Discussion

Whooping cough is a highly contagious disease that remains a global health concern despite being preventable through vaccination. While it poses the greatest risk to unvaccinated infants, it increasingly affects adults, particularly those over 50 [5]. Since the introduction of pertussis vaccines in the 1950s, along with widespread immunisation programmes, childhood mortality has significantly declined [6]. However, although childhood vaccination is routine in many countries, immunisation of older adults is less commonly practiced [5].

According to data from Public Health England, in 2019, 32% of all reported cases of pertussis (whooping cough) occurred in adults aged 45 years and older. In adolescents and adults, pertussis may present with atypical symptoms, often limited to a mild or persistent cough. In some cases, it can cause more severe effects, including weight loss, headaches, sneezing fits, sleep disruption, sore throat, sinus pain, and excessive sweating [7]. Asymptomatic or mildly symptomatic adults can unknowingly transmit the infection to infants who are not yet fully immunised [8]. 

Immunity following natural infection is generally considered more robust than that induced by vaccination, and pertussis is no exception [9]. However, protection against Bordetella pertussis, whether acquired through infection or vaccination, wanes over time [10]. Natural immunity typically lasts between 4 and 20 years, while vaccine-induced protection is shorter, lasting approximately 4 to 12 years [11]. Studies also suggest that immunity declines more rapidly after immunisation with the acellular pertussis (aP) vaccine compared to the whole-cell pertussis (wP) vaccine [12]. The wP vaccine offers more durable protection because it elicits stronger Th1 and Th17 responses, as well as tissue-resident memory T cells, which are crucial for bacterial clearance and the prevention of transmission [13]. 

Optic neuropathy is common and has many aetiologies [14]. A quick onset is characteristic of inflammatory, demyelinating, ischemic, and traumatic causes. A slower progression suggests compressive, hereditary or toxic/nutritional factors. The hallmark clinical manifestations of optic neuropathy include visual field defects, dyschromatopsia, and an abnormal papillary response. There are additional investigations that can aid in the diagnosis of optic neuropathy. Neuroimaging of the brain and orbit is vital in numerous cases of optic neuropathy, particularly those that are demyelinating or compressive.

Upon our literature search in PubMed, Embase and Google Scholar, we could not find documented studies and case reports suggesting the link between Pertussis infection and bilateral optic neuropathy leading to blindness. We therefore hypothesize a possible link between the two and declare this case as an index presentation needing further studies.

Our study has limitations. It is a case report and has no studies to support our hypothesis. Our patient lost vision completely; hence, there is no suggestion of treatment in this hypothesized link. More studies are needed to validate our findings.

## Conclusions

Pertussis infections in adults and bilateral optic neuropathy are rare entities. Our case highlights a potential link between the two. We propose that more studies are needed to evaluate the link between pertussis infection and bilateral optic neuropathy as our patient had poor prognosis despite steroid therapy. A multi-disciplinary team approach by acute internal medicine, neurology and ophthalmology is key in managing such challenging cases.
